# Blockade of pathological retinal ganglion cell hyperactivity improves optogenetically evoked light responses in rd1 mice

**DOI:** 10.3389/fncel.2015.00330

**Published:** 2015-08-25

**Authors:** John M. Barrett, Patrick Degenaar, Evelyne Sernagor

**Affiliations:** ^1^Faculty of Medical Sciences, Institute of Neuroscience, Newcastle UniversityNewcastle-upon-Tyne, UK; ^2^Faculty of Science, Agriculture and Engineering, School of Electrical and Electronic Engineering, Newcastle UniversityNewcastle-upon-Tyne, UK

**Keywords:** retinal degeneration, retinal prosthesis, optogenetics, spontaneous activity, meclofenamic acid, flupirtine, 18-beta-glycyrrhetinic acid

## Abstract

Retinitis pigmentosa (RP) is a progressive retinal dystrophy that causes visual impairment and eventual blindness. Retinal prostheses are the best currently available vision-restoring treatment for RP, but only restore crude vision. One possible contributing factor to the poor quality of vision achieved with prosthetic devices is the pathological retinal ganglion cell (RGC) hyperactivity that occurs in photoreceptor dystrophic disorders. Gap junction blockade with meclofenamic acid (MFA) was recently shown to diminish RGC hyperactivity and improve the signal-to-noise ratio (SNR) of RGC responses to light flashes and electrical stimulation in the rd10 mouse model of RP. We sought to extend these results to spatiotemporally patterned optogenetic stimulation in the faster-degenerating rd1 model and compare the effectiveness of a number of drugs known to disrupt rd1 hyperactivity. We crossed rd1 mice with a transgenic mouse line expressing the light-sensitive cation channel channelrhodopsin2 (ChR2) in RGCs, allowing them to be stimulated directly using high-intensity blue light. We used 60-channel ITO multielectrode arrays to record ChR2-mediated RGC responses from wholemount, *ex-vivo* retinas to full-field and patterned stimuli before and after application of MFA, 18-β-glycyrrhetinic acid (18BGA, another gap junction blocker) or flupirtine (Flu, a Kv7 potassium channel opener). All three drugs decreased spontaneous RGC firing, but 18BGA and Flu also decreased the sensitivity of RGCs to optogenetic stimulation. Nevertheless, all three drugs improved the SNR of ChR2-mediated responses. MFA also made it easier to discern motion direction of a moving bar from RGC population responses. Our results support the hypothesis that reduction of pathological RGC spontaneous activity characteristic in retinal degenerative disorders may improve the quality of visual responses in retinal prostheses and they provide insights into how best to achieve this for optogenetic prostheses.

## 1. Introduction

Retinitis pigmentosa (RP) is a retinal dystrophy characterized by progressive photoreceptor death, starting with the rods, causing night-blindness and a loss of peripheral vision, followed eventually by total blindness as the cones start to degenerate as well (Heckenlively et al., 1988; Berson, 1993; Hartong et al., 2006). It has a global prevalence of approximately one in 4000 (Hartong et al., 2006). At present, the only clinically available treatment capable of restoring vision in RP (as opposed to slowing or halting progression of visual loss) is retinal prosthesis. Current retinal prostheses use implanted electrodes in combination with photovoltaics (Mathieson et al., 2012; Stingl et al., 2013) or an external light sensor (Dorn et al., 2013) to deliver patterned electrical stimulation to the retina and evoke a visual percept (Margalit et al., 2002), but thus far such devices have only managed to restore crude vision (Dorn et al., 2013; Stingl et al., 2013). Possible reasons for this include limited resolution due to the low number of electrodes (presently 60–1500, Dorn et al., 2013; Stingl et al., 2013), lack of control over the spatial spread of charge, lack of cell-type specificity and the ability to provide excitatory but not inhibitory stimulation (Barrett et al., 2014). Optogenetics, in which neurons are engineered to express light-sensitive ion channels to enable optical control of membrane potential (Boyden et al., 2005; Bernstein and Boyden, 2011; Deisseroth, 2011; Fenno et al., 2011), may be able to overcome many of these limitations of electrical prostheses. Thus, the past decade has seen considerable progress in the development of optogenetic retinal prostheses, in which surviving inner retinal neurons are made light sensitive to restore vision (for review, see Busskamp and Roska, 2011; Busskamp et al., 2012; Cepko, 2012; Sahel and Roska, 2013; Barrett et al., 2014).

However, an often overlooked problem of retinal degenerations in retinal prosthetic research is the extensive remodeling of the inner retina that follows photoreceptor death (Marc et al., 2003; Jones and Marc, 2005; Marc et al., 2007), which results in slow local field potential (LFP) oscillations in the inner retina and rhythmic bursting of retinal ganglion cells (RGCs). This pathological hyperactivity is observed in numerous animal models of photoreceptor dystrophy, including the *rd1* mouse (Stasheff, 2008), the *rd10* mouse (Goo et al., 2011; Stasheff et al., 2011), the CRX mouse (Soto et al., 2012; Maccione et al., 2014), and the P23H rat (Sekirnjak et al., 2009). In the *rd1* mouse, lack of photoreceptor input results in the AII amacrine cells becoming tonically hyperpolarized, revealing intrinsic, low-frequency (approximately 10 Hz) oscillations in these cells (Choi et al., 2014) that then spread via gap junctions through the AII-ON bipolar cell network (Menzler and Zeck, 2011; Trenholm et al., 2012), resulting in RGC bursting. Similar oscillations are observed in wild-type mouse after blocking photoreceptor to bipolar cell synapses (Trenholm et al., 2012; Choi et al., 2014). Oscillations in the *rd10* mouse are slightly lower frequency than the *rd1* but are pharmacologically similar (Biswas et al., 2014), suggesting a similar underlying mechanism. In summary, low frequency oscillations and increased spontaneous RGC firing are common to numerous mouse models of photoreceptor dystrophy and appear to share a common mechanism.

As a result, any signal delivered prosthetically may be more difficult to distinguish against this background of higher and more bursty spontaneous firing of RGCs in the degenerate retina. Recently, Toychiev et al. (2013) demonstrated that blocking the pathological spontaneous activity with the gap junction blocker meclofenamic acid (MFA) improves the signal-to-noise ratio (SNR) of surviving photoreceptor responses and responses to electrical stimulation in the *rd10* mouse retina (Toychiev et al., 2013; Ivanova et al., 2015). Here, we set out to determine if the same principle works in other models of retinal degeneration, specifically the fast-degenerating *rd1* model, and for optogenetic stimulation. Additionally, Toychiev et al. (2013) considered only responses to full-field illumination, so we sought to investigate the effects of reducing spontaneous activity on responses to spatiotemporally patterned stimulation using a novel 256-pixel microLED (μLED) array (Grossman et al., 2010; Al-Atabany et al., 2013). Finally, we tested a number of drugs with different mechanisms of action to ascertain whether specific blockade of gap-junctions is sufficient and necessary to block these pathological oscillations or whether general reduction in spontaneous activity can achieve the same results.

## 2. Materials and methods

### 2.1. Experimental animals

All experimental procedures were approved by the local ethics committee at Newcastle University and were conducted in line with the UK Home Office Animals (Scientific Procedures) Act 1986. C3H/HeNHsd mice (also known as *rd1*) were purchased from Harlan Laboratories (Indianapolis, USA). These mice express the naturally-occurring *Pde6b*^*rd*/*rd*^ mutant allele of rod phosphodiesterase, which causes rapid rod death followed by secondary cone loss, with virtually no surviving photoreceptors by postnatal day 36 (P36) (Carter-Dawson et al., 1978). We crossbred these with B6.Cg-Tg(Thy1-COP4/EYFP)9Gfng/J mice from the Jackson Laboratory (Bar Harbor, USA), which express the light-sensitive cation channel *channelrhodopsin-2* (ChR2) (Nagel et al., 2003; Boyden et al., 2005) in a number of cell types through the central nervous system, including RGCs (Arenkiel et al., 2007; Thyagarajan et al., 2010). All experimental animals were at least second generation crosses (*ChR2rd1*) and were homozygous for *Pde6b*^*rd*/*rd*^ and at least hemizygous for ChR2.

### 2.2. Electrophysiology

Mice were killed by cervical dislocation and their eyes quickly enucleated and placed into room-temperature artificial cerebrospinal fluid (aCSF) containing (in mM) 118 NaCl, 25 NaHCO_3_, 1 NaH_2_PO_4_, 3 KCl, 1 MgCl_2_, 2 CaCl_2_, and 10 glucose, equilibrated with 95% O_2_ and 5% CO_2_ for retinal dissection. The isolated retina was placed wholemount, RGC layer facing down, onto a 60-channel indium tin oxide multielectrode array (MEA; Multichannel Systems, Reutlingen, Germany). A small piece of polyester membrane filter (5 μm pores) (Sterlitech, Kent, WA, USA) and a diamond- or ring-shaped metal weight (Warner Instruments, Hamden, CT, USA) were placed on the retina to improve coupling between the tissue and the electrodes. Once in the MEA chamber, the retina was kept at 32°C and continuously perfused with aCSF at 1–2 ml/min. The retina was allowed to settle for 2 h before any recordings were taken. Electrophysiological activity was recorded at a sampling rate of 25 kHz using MC_Rack software (Multichannel Systems).

### 2.3. Pharmacology

We used three drugs in our experiments. MFA is a gap-junction blocker (Pan et al., 2007; Veruki and Hartveit, 2009) that has previously been shown to improve SNR of light and electrical responses of RGCs in dystrophic retinas (Toychiev et al., 2013; Ivanova et al., 2015). Flupirtine is a Kv7 potassium channel opener (Martire et al., 2004; Wladyka and Kunze, 2006; Yeung et al., 2007) that has recently been shown to block spontaneous activity in degenerate retinas by dampening intrinsic oscillations in the AII amacrine cell (Trenholm et al., 2012; Choi et al., 2014). MFA also affects the same potassium channel (Peretz et al., 2005; Yeung et al., 2007), so we also tested a second gap-junction blocker, 18-β-glycyrrhetinic acid (18BGA) (Davidson et al., 1986; Syed et al., 2004; Pan et al., 2007; Sun et al., 2008), that to our knowledge has no effect on the Kv7 channel. Both 18BGA and the closely related compound carbenoxolone have been shown to dampen *rd1* spontaneous activity (Menzler and Zeck, 2011; Trenholm et al., 2012). MFA and 18BGA were purchased from Sigma-Aldrich (St Louis, USA); Flu from Abcam (Cambridge, UK). Recordings were taken in control conditions, once at each drug concentration (10, 20, 40, and 80 μM) and again after washout. Drugs were added to the aCSF: separate reservoirs were maintained for each drug concentration. Thirty to forty-five minutes were allowed for the drug to take effect after each increase in concentration. Washout was between 1 and 3 h. Only one drug was used on any given retina.

### 2.4. Optogenetic stimulation

Stimuli were presented using a custom-made, 256-pixel, Gallium Nitride microLED (μLED) array (Grossman et al., 2010; Al-Atabany et al., 2013). Light from the μLED array was projected through the camera port of an Olympus IX-71 inverted microscope (Olympus, Tokyo, Japan) and focused onto the RGC layer using a 2 × objective. Each pixel covered an area of approximately 62.5 μm on the retina and hence the whole array covered an area of roughly 1 mm^2^. The image of the array was positioned so as to cover either the central 6 × 6 electrodes or the set of electrodes showing the strongest electrophysiological activity. Three sets of stimuli were used: full-field flashes with durations of 5, 10, 25, 50, 75, or 100 ms presented every 2 s; flashing squares of 1 × 1, 2 × 2, or 4 × 4 pixels presented for 100 ms each; and bars of width 2 pixels moving in the 8 cardinal and ordinal compass directions at speeds of one pixel every 50 or 100 ms (1250 or 625 μm/s) presented every 4 s. All stimuli were presented in control conditions and at the highest drug concentration. Additionally, the full-field flashes were presented at all intermediate drug concentrations and after washout. Each set of stimuli at a given drug concentration was presented in randomized blocks: 20 blocks for the full-fields and 10 blocks each for the flashing squares and moving bars. The total power transmitted from the μLED array to the retina was measured as 25.5 μW using a Newport 1918-R optical power meter (Newport Spectra-Physics Ltd, Didcot, UK) equipped with a Newport 818-UV/DB photodiode. Averaged over the entire (approximately 1 mm^2^) image this corresponds to an irradiance of 25.5 μW/mm^2^. In practice due to the separation between individual LEDs there will be regions of zero irradiance and regions of higher irradiance (equal to the average irradiance divided by the fill factor). In one experiment, the power output was accidentally set to 21.0 μW, but the results from this experiment were not qualitatively different from the rest and so they were included in all analyses presented here.

### 2.5. Analysis

To analyse LFP oscillations, raw MC_Rack data was imported into Matlab (The MathWorks, Natick, USA) using the FIND toolbox. Roughly 1 min of data (specifically 83.88 s, i.e., the number of samples (2^21^ = 2097152) equal to the next integer power of two greater than 1 min of recording at 25 kHz) was extracted from the middle of each spontaneous activity recording and its power spectrum computed. The oscillation strength was quantified as the area under the peak of the power spectrum over the full width at half maximum.

To analyse spontaneous firing and spiking responses to optogenetic stimulation, spikes were first extracted by high-pass filtering the data in MC_Rack with a cut-off of 300 Hz and then applying a voltage threshold. The threshold was set for each channel for each retina independently as seven standard deviations below a baseline 60 s recording from the empty MEA at the beginning of each experiment and then adjusted manually to ensure as many spikes were detected as possible while minimizing noise. Spike waveforms comprising 16 samples before and 32 samples after each threshold crossing were extracted and imported into Offline Sorter (Plexon, Dallas, USA) for spike sorting. Automatic spike sorting was performed using T-distribution Expectation-Maximization (Shoham et al., 2003), followed by manual inspection to ensure accuracy of sorting.

#### 2.5.1. Detecting optogenetically responsive cells

Not all RGCs in these retinas express ChR2, hence responses to the longest-duration full-field flashes were used to detect RGCs that responded to optogenetic stimulation. First, a spontaneous firing distribution was bootstrapped by dividing the 1 s periods before each flash into 100 ms bins (equal to the length of the longest flash), computing the median number of spikes in 10 randomly selected bins and repeating this procedure 10,000 times. This distribution was used to assign one-sided *p*-value to the median number of spikes fired by a cell in response to 100 ms flashes, under the null hypothesis that the cell does not respond to stimulation. Those cells within a recording having *p* < 0.05 after false-discovery rate correction (Yoav and Hochberg, 1995) were deemed responsive. To avoid double-dipping (Kriegeskorte et al., 2009), only odd-numbered trials of the full-field flashes were used to detect responsive cells and only even-numbered trials were used to calculate thresholds and SNR (see below).

#### 2.5.2. Stimulation threshold and signal-to-noise ratio

For each cell that was responsive in both control conditions and at the highest drug concentration, the stimulation threshold and SNR were calculated as follows. The response probability as a function of flash duration was calculated by counting the number of trials on which the number of spikes fired in the 100 ms following the onset of a flash exceeded the median number of spikes fired in any 100 ms period of spontaneous activity. This response probability function was fit with a sigmoid function using the lsqcurvefit function in Matlab:

$$\begin{array}{r} p\left(t\right)=\frac{1}{1+e^{-\frac{t-b}{a}}} \end{array}$$

The parameter *b* gives flash duration with a 50% probability of evoking a response, which was taken as the threshold for a given cell. The signal-to-noise ratio (SNR) is commonly defined as the mean of the signal divided by the standard deviation of the noise. The signal we are interested in here is those spikes evoked by the μLED flash, but it is impossible to distinguish stimulus-evoked spikes from spontaneous spikes that happened to be fired immediately after a stimulus, hence we estimated the SNR for a given cell as

$$\begin{array}{r} SNR=\frac{\mu_{signal+noise}-\mu_{noise}}{\sigma_{noise}} \end{array}$$

Where μ_*signal*+*noise*_ is the mean number of spikes (spontaneous and evoked) fired in the 100 ms following any even-numbered flash, μ_*noise*_ is the mean number of spikes fired in any 100 ms bin of spontaneous activity and σ_*noise*_ is the standard deviation of the number of spikes fired in any 100 ms bin of spontaneous activity. Some RGCs in each recording only fired immediately following a flash and so their SNR was immeasurably high: these cells were assigned an SNR of infinity.

#### 2.5.3. Spike triggered averaging and receptive field measurement

Responses to the 2 × 2 and 4 × 4 pixel flashed squares were used to construct spike-triggered averages (STAs) for each cell (the 1 × 1 pixel flashes were found to produce very weak responses, if any, and so were excluded from the STA). A 16 × 16 matrix of zeros—one entry per μLED pixel—was instantiated for each cell. For each frame of each stimulus, the number of spikes fired by that cell during presentation of that stimulus frame was added to those matrix entries corresponding to the pixels that were on during that frame. Finally, each entry in the matrix was divided by the number of stimulus frames in which the corresponding pixel was on. Each STA was fit with a raised two-dimensional Gaussian function using Matlab's lsqcurvefit function:

$$\begin{array}{lll} a & =\frac{{\text{cos}}^{2}\theta }{2\sigma_{x}^{2}}+\frac{{\text{sin}}^{2}\theta }{2\sigma_{y}^{2}}\; & \; \end{array}$$

$$\begin{array}{lll} b & =-\frac{\text{sin}2\theta }{4\sigma_{x}^{2}}+\frac{\text{sin}2\theta }{4\sigma_{y}^{2}}\; & \; \end{array}$$

$$\begin{array}{lll} c & =\frac{{\text{sin}}^{2}\theta }{2\sigma_{x}^{2}}+\frac{{\text{cos}}^{2}\theta }{2\sigma_{y}^{2}}\; & \; \end{array}$$

$$\begin{array}{lll} RF\left(x,y\right) & =Ae^{-\left(a{\left(x-x_{c}\right)}^{2}+2b\left(x-x_{c}\right)\left(y-y_{c}\right)+c{\left(y-y_{c}\right)}^{2}\right)}+B\; & \; \end{array}$$

The receptive field radius was then calculated as the geometric mean of the semimajor and semiminor axes of the 1-SD contour of the fitted Gaussian, i.e., $r=\sqrt{\sigma_{x}\sigma_{y}}$. This corresponds to the radius of a circle having the same area as the fitted receptive field. The receptive field aspect ratio was calculated as σ_y_ ∕ σ_x_.

#### 2.5.4. Moving bars

Each cell's spike train during the presentation of the moving bar stimuli was convolved with a one-dimensional Gaussian function with a standard deviation of 25 ms to estimate the instantaneous firing rate (IFR). The time to peak firing on each trial was calculated as the time between the appearance and disappearance of the bar that the IFR reached its maximum value, relative to bar onset. If a cell only fires when a sufficient amount of light falls within its receptive field, this time to peak firing should provide a reliable estimate of the point along the bar's trajectory at which it entered the cell's receptive field. If the cell's receptive field is not exactly in the middle of the array, this will also give some information as to the direction of travel of the bar.

To quantify how well the population of optogenetically sensitive cells in a given retina encodes stimulus direction, a modified naive Bayesian classifier was trained to determine bar direction. Given a set of observations **r** = {*r*_1_, …, *r*_*N*_} that can belong to a class *s* ∈ {*s*_1_, …, *s*_*k*_}, a Bayesian classifier attempts to assign the response to the most likely class ŝ using Bayes' rule, assuming that the random variables (or “features”) that comprise the observation are conditionally independent:

$$\hat{s}=\underset{s}{\arg\text{ max}}\;p(s|r)=\underset{s}{\arg\text{ max}}\frac{\;p(r|s)p(s)}{p(r)}=\underset{s}{\arg\text{ max}}\;p(s)\prod_{i=1}^{N}p(r_{i}|s)$$

(Note that the denominator is irrelevant because it is the same for all classes.) Here, each feature is the response of one RGC (quantified as time to peak firing) and each class is the direction of the moving bar that evoked that response. All RGCs that responded in control or drug conditions were included in the classifier. A typical naive Bayesian classifier assumes each *p*(*r*_*i*_|*s*) follows a Gaussian distribution. However, on some trials, particularly in the drug condition, cells do not fire and therefore the time to peak firing is not defined, so we treat it as infinite. To account for this, each RGC's response conditioned on the stimulus direction was assumed to be a mixture of a discrete, Bernoulli-distributed random variable (response or no response) and a continuous, Gaussian-distributed random variable (time to peak firing, if finite):

$$p(r|s)=\{\begin{array}{ll} q_{s} & \text{ if }r=\infty \\ \left(1-q_{s})g(r;\mu_{s},\sigma_{s}\right) & \text{ if }r<\infty \end{array}$$

where *r* is the time to peak firing of the cell, *s* is the bar direction, *g*(*r*; μ_*s*_, σ_*s*_) is the probability density function of a random variable following a Gaussian distribution with mean μ_*s*_ and variance $\sigma_{s}^{2}$, and 0≤*q*_*s*_ ≤ 1. It is trivial to show that, given a sample {*x*_1_, …, *x*_*m*_, *y*_1_, …, *y*_*n*_} (where *x*_*i*_ = ∞ for all 1≤*i*≤*m*) of observations from the above distribution, the maximum likelihood estimator (MLE) of *q*_*s*_ is $\frac{m}{m+n}$ and the MLEs of μ_*s*_ and σ_*s*_ are the mean and standard deviation of {*y*_1_, …, *y*_*n*_}, respectively. *p*(*s*) followed a uniform distribution with eight values, i.e., *p*(*s*) = 1∕8 for all *s*.

Each set of bar responses was partitioned into a training set and a test set. The training set was used to estimate $p(r|s)=\prod_{i=1}^{N}p(r_{i}|s)$. The decoder performance is the percentage of test trials in which ŝ equals the stimulus that was actually presented. This is repeated for multiple instantiations of training and test sets to obtain an average decoder performance. We used leave-one-out cross-validation, in which the size of the test set is always one, the training set is every trial apart from the test trial, and each trial is used as the test set exactly once.

#### 2.5.5. Statistical analysis

All analyses presented here are non-parametric repeated measures designs with drug concentration as the within-subjects factor, so the Friedman test was used. Different retinas were used as the blocking factor except where noted in the text. Where multiple measurements were taken from a single retina (i.e., multiple channels or multiple cells), the median value for each retina was used in the analysis. As the Friedman test operates on ranks, infinite SNR values were assigned the mean of the rank they would have been assigned had they been finite, monotonically increasing values greater than any measurable SNR value. Due to the uneven number of retinas used for each drug for some analyses, separate tests were used for each drug and so no direct statistical comparisons between drugs are presented here (although the Friedman test does not assess the significance of between-subject effects anyway). All *p*-values are reported uncorrected, but all those significant at the *p* < 0.05 level remained significant after Holm-Bonferroni correction for multiple comparisons with an α level of 0.05.

## 3. Results

### 3.1. Spontaneous activity and full-field stimulation

**Figure 1:**
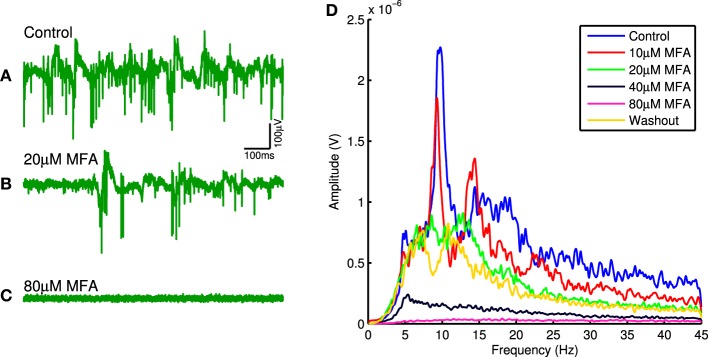
**(A–C)** Raw electrode trace on one channel for an example retina in control conditions **(A)**, with 20 μM MFA **(B)**, and with 80 μM MFA **(C)**. Note the decrease in both oscillations and level of spontaneous firing as the drug concentration increases. **(D)** Power spectra recorded on the same channel in control conditions, at each drug concentration and after washout. Notice the overall decrease in LFP power as the drug concentration increases and the recovery upon washout.

**Figure 2:**
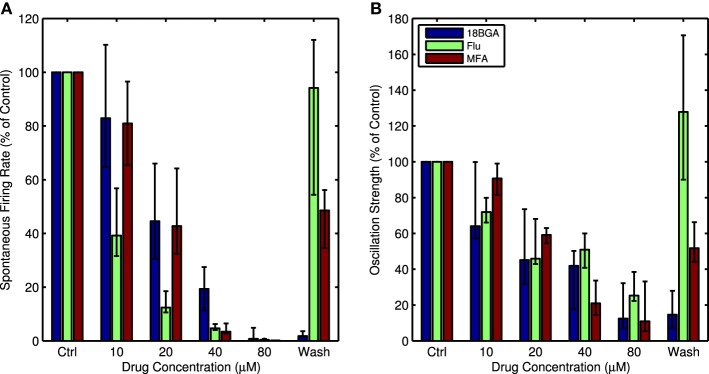
**(A)** Spontaneous firing rate as a percentage of control conditions for each concentration of all three drugs, averaged over all channels with recorded spikes and all retinas. **(B)** Oscillation strength as a percentage of control conditions for each concentration of all three drugs, averaged over all recorded channels and all retinas. For both figures, error bars show the interquartile range (IQR).

Figure 1 shows raw traces recorded from one channel in control conditions and in the presence of 20 μM and 80 μM MFA, as well as the power spectra from this same channel at each drug concentration. There is a clear decrease in oscillatory activity and spontaneous firing as the drug concentration increases. This pattern held across all retinas for all three drugs tested. Each drug significantly reduced the strength of LFP oscillations (Friedman test: 18BGA *n* = 7, *p* = 2.5 × 10^−5^; Flu *n* = 7, *p* = 0.0001; MFA *n* = 7, *p* = 6 × 10^−6^) and spontaneous RGC firing (Friedman test: 18BGA *n* = 7, *p* = 1.1 × 10^−5^; Flu *n* = 7, *p* = 6 × 10^−6^; MFA *n* = 7, *p* = 1.5 × 10^−5^). Figure 2 shows the effect of each drug on oscillation strength and spontaneous firing rate relative to control conditions averaged across all recorded channels for all retinas. Flu has a stronger effect at low concentrations, consistent with a previous study showing that 10 μM Flu blocks spontaneous activity (Choi et al., 2014), but for all three drugs spontaneous firing is almost completely abolished at 80 μM. There is substantial recovery of oscillations and spontaneous firing after washout of Flu and MFA, consistent with previous reports (Menzler and Zeck, 2011; Trenholm et al., 2012; Choi et al., 2014), but not with 18BGA, the effects of which are known to be irreversible at high concentrations (Rozental et al., 2001).

**Figure 3:**
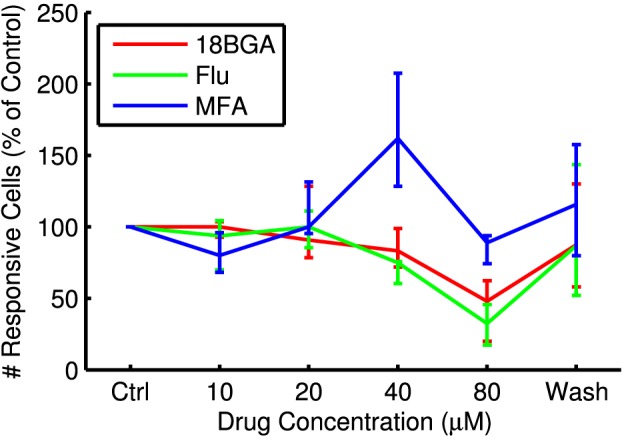
**Number of cells responding to the longest μLED array flash at each drug concentration as a percentage of control conditions, averaged over all retinas**.

**Figure 4:**
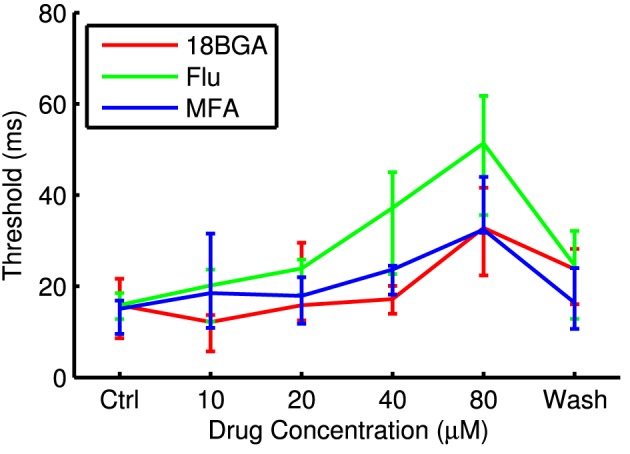
**Threshold flash duration for optogenetically sensitive cells at each drug concentration**. Data points are median over all retinas of the median threshold of all cells that respond in both control conditions and at 80 μM drug. Error bars are IQR for all retinas.

Lowering spontaneous activity has the potential to improve SNR, but not if it comes at the expense of the ability to stimulate RGCs optogenetically. Figure 3 shows the number of cells that respond to the longest full-field μLED array flash at each drug concentration, relative to control conditions. All three drugs significantly affected the number of responsive cells (18BGA *n* = 7, *p* = 0.0037; Flu *n* = 7, *p* = 0.0019; MFA *n* = 7, *p* = 0.0058). 18BGA and Flu both caused a dose-dependent decrease in the number of responsive cells (in one experiment for each, there were no responding cells left at 80 μM and so these experiments were excluded from the threshold and SNR analyses). The pattern for MFA is more complicated: at most concentrations, the number of responsive cells was similar to control conditions, but the number of responsive cells appears to increase at 40 μM before returning to baseline at 80 μM. Figure 4 shows the threshold flash duration for those cells that responded to optogenetic stimulation in both control conditions and the highest drug concentration. All three drugs appear to cause a dose-dependent increase in stimulation thresholds (18BGA *n* = 6, *p* = 0.0097; Flu *n* = 6, *p* = 0.0002; MFA *n* = 7, *p* = 0.0087). Taken together, these results suggest that 18BGA and Flu hinder optogenetic stimulation of RGCs, whereas MFA has a mixed effect, increasing the number of responsive cells at the expense of increasing the stimulation threshold.

**Figure 5:**
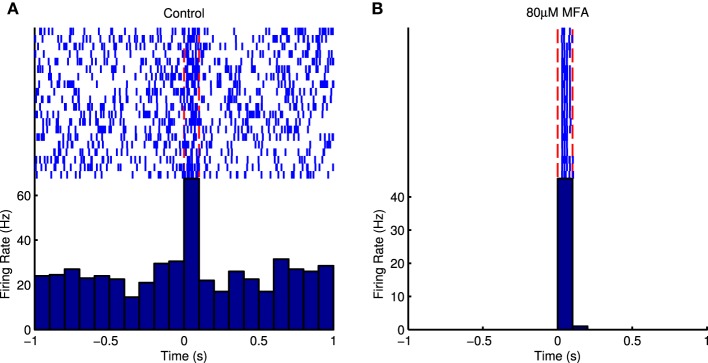
**(A)** Raster plot and PSTH of an example cell in response to 100 ms full-field μLED flashes in control conditions. The light is on between the red dotted lines. It is difficult to distinguish the evoked responses from the spontaneous bursts that occur randomly between stimuli. **(B)** Raster plot and PSTH of the same cell in response to the same stimulus in the presence of 80 μM MFA. The spontaneous activity is abolished and the evoked response is very distinct.

**Figure 6:**
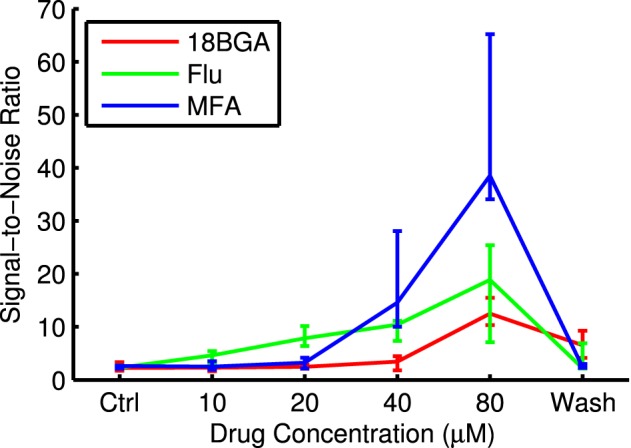
**Signal-to-noise ratio for responses to the longest μLED array flash at each drug concentration**. Data points are median over all retinas of the median SNR of all cells that respond in both control conditions and at 80 μM drug and had measurable SNR values. Error bars are IQR for all retinas.

Figure 5 shows raster plots and PSTHs from an example cell in response to the longest flash in control conditions and at the highest drug concentration. It is very difficult to distinguish the cell's response from the high level of spontaneous activity in control conditions, but the response to light is very distinct once the spontaneous activity is abolished. Figure 6 shows SNR as a function of drug concentration for those cells that responded in control conditions and at 80 μM and had measurable SNR values. Note that some responsive cells did not fire any spontaneous spikes during the recording and hence we could not estimate their SNR and so the values in the figure are underestimates. These cells were assigned an SNR of infinity for the purpose of statistical analysis (see Section 2.5.5). In four retinas each for Flu and MFA, over half the responsive cells had infinite SNR at 80 μM. All three drugs significantly increased SNR (Friedman test, flash duration as blocking factor: 18BGA *n* = 6, *p* = 0.0004; Flu *n* = 6, *p* = 1.2 × 10^−7^; MFA *n* = 7, *p* = 3.7 × 10^−7^).

### 3.2. Spatiotemporally patterned stimulation

**Figure 7:**
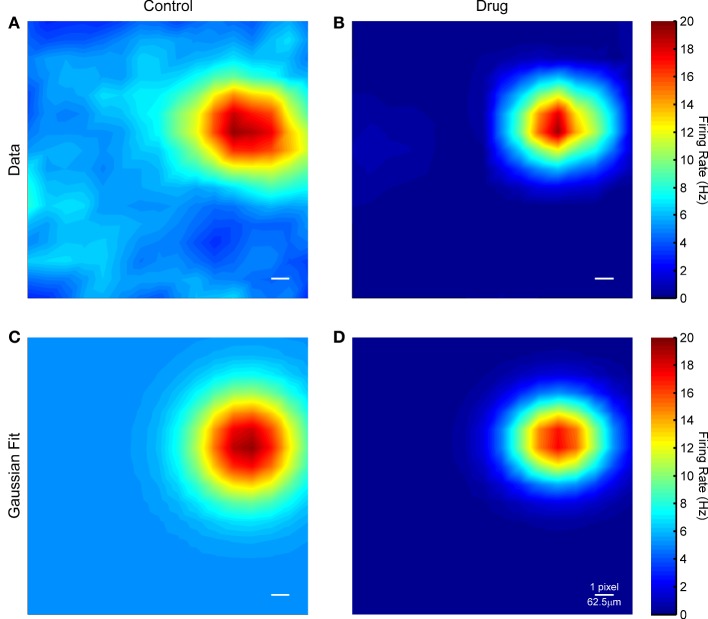
**(A)** Spike triggered average for an example RGC in control conditions. **(B)** Spike triggered average for the same cell in the presence of 80 μM MFA. **(C,D)** Gaussian fits to the data in **(A,B)**. The scale bar in each panel is one μLED array pixel or approximately 62.5 μm in length.

Figure 7 shows example STAs recovered from responses to flashing squares in control conditions and at a drug concentration of 80 μM. In both cases, a clear hotspot is observed, presumably corresponding to those pixels that overlap the RGC, but the surrounding pixels are slightly noisier in control conditions. This pattern was similar across all cells for which responses to flashing squares were successfully recorded. The median receptive field diameter was 245.2 μm in control conditions and 219.8 μm in the drug condition, but this difference was not significant. In both control and drug conditions the recovered RFs were mostly roughly circular, with a median aspect ratio of 1.17. ChR2 in these retinas is expressed throughout the cell (data not shown, see also Thyagarajan et al., 2010), including soma, dendrites, and axons. The size and shape of the ChR2 receptive fields is consistent with ChR2 activation in the soma and dendrites being primarily responsible for spike generation, rather than axonal stimulation, even though the light had to pass through the nerve fiber layer before reaching the RGCs.

**Figure 8:**
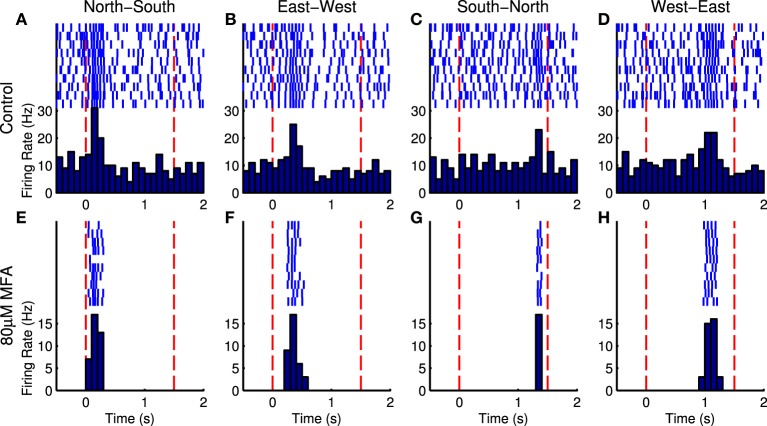
**Raster plots and PSTHs for an example in response to bars moving in the four cardinal compass directions at 625 μm/s in control conditions (A–D) and in the presence of 80 μM MFA (E–H)**. The bar appears at the first red dotted line, sweeps across the array, and disappears at the second red dotted line. On a trial-by-trial basis, random spontaneous bursts may corrupt the estimate of time to peak firing and hence when the bar enters the cell's receptive field. After blocking spontaneous activity, the cell only fires when the bar passes over its receptive field. This cell was located at the north-east corner of the array, so it reaches its peak firing rate very soon after bar onset when the bar starts in the north or east of the array **(A,B,E,F)**, but very late when the bar originates in the south or west **(C,D,G,H)**.

**Figure 9:**
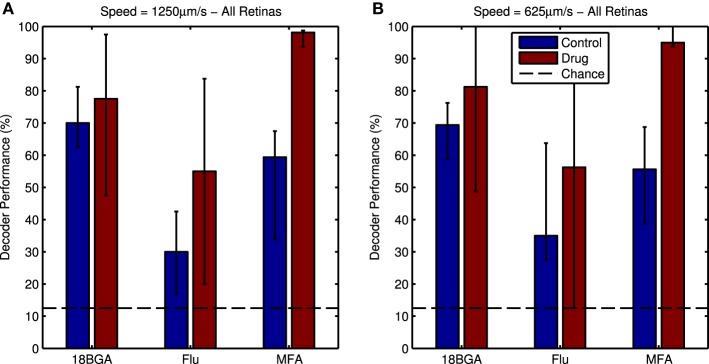
**(A)** Bayesian decoder performance for responses to the 1250 μm/s bars in control conditions (blue bars) and in the presence of 80 μM of each drug (red bars). Decoder performance is much higher in the presence of the drug. Dotted line indicates chance level performance. **(B)** the same plot for the 625 μm/s bars. For both panels, data points are medians over all retinas and error bars are IQRs.

Figure 8 shows responses of an example cell to four directions of the moving bar stimulus, in control and drug conditions. It is difficult to discern the response to the bar in the presence of spontaneous hyperactivity, but there is a clearly distinguishable peak in the cell's firing when the bar enters its receptive field in the drug condition. Nevertheless, the Bayesian classifier was modestly successful in decoding stimulus direction in control conditions, achieving correct decoding roughly 40–70% of the time on average (Figure 9, blue bars). After applying the drug, the decoder performance is improved, with the classifier decoding the stimulus direction correctly 70–100% of the time on average (Figure 9, red bars). This difference was significant for MFA (Friedman's test, bar speed as blocking factor: *n* = 7, *p* = 0.0001) but not Flu (*n* = 7, *p* = 0.44) or 18BGA (*n* = 7, *p* = 0.57).

## 4. Discussion

### 4.1. Blocking spontaneous activity improves snr regardless of the the mechanism of action

It has been shown recently that blocking spontaneous activity with MFA improves SNR of residual photoreceptor responses and responses to electrical stimulation in the *rd10* mouse (Toychiev et al., 2013; Ivanova et al., 2015). Our results show that this principle also holds for optogenetic stimulation in the *rd1* mouse. Spontaneous activity in degenerate retinas is believed to originate in the AII amacrine cell (Trenholm et al., 2012; Choi et al., 2014) and propagate through the retinal network via gap junction coupling between AII amacrine cells and other cell types, such as ON-cone bipolar cells (Menzler and Zeck, 2011; Trenholm et al., 2012; Yee et al., 2012). These oscillations can be strengthened or dampened by altering the potassium conductance of AII cells (Choi et al., 2014), and prevented from spreading by blocking gap junctions (Menzler and Zeck, 2011; Trenholm et al., 2012; Toychiev et al., 2013; Biswas et al., 2014).

MFA is both a gap-junction blocker (Pan et al., 2007) and a modulator of Kv7 potassium channels (Peretz et al., 2005; Yeung et al., 2007), so it is not clear which of these mechanisms is responsible for its effects on spontaneous hyperactivity. In principle, it should not matter for improving SNR, but to confirm this we tested two additional drugs: Flu, which is a powerful Kv7 potassium channel opener (Martire et al., 2004; Wladyka and Kunze, 2006; Yeung et al., 2007) that has recently been shown to block *rd1* spontaneous activity (Choi et al., 2014); and 18BGA, another gap junction blocker (Davidson et al., 1986). As expected, all three drugs significantly reduced spontaneous firing and improved SNR of optogenetic responses, at least for those cells that were still sensitive to ChR2 stimulation. These results demonstrate that being able to suppress the pathological spontaneous activity, rather than the specific approach to do so, is the key requisite to enhance the SNR during electrical or optogenetic stimulation. In principle, any other means of blocking spontaneous activity, for example blocking synaptic input onto RGCs (Borowska et al., 2011; Menzler and Zeck, 2011; Trenholm et al., 2012; Biswas et al., 2014), should also improve the SNR of optogenetic responses, although we have not tested this.

### 4.2. Effects on stimulation efficiency of chr2 rgcs

Increasing the SNR of individual cells by decreasing spontaneous activity may not lead to improved prosthetic signal transmission through the retina if the method of decreasing spontaneous activity also leads to fewer cells responding to stimulation. Hence, we measured the number of cells responding to stimulation and their stimulation threshold as a function of drug concentration. Both 18BGA and Flu appear to lead to a dose-dependent decrease in the number of cells responding and an increase in stimulation thresholds. In the case of Flu, this may be because it acts by increasing the conductance of the Kv7 potassium channel (Peretz et al., 2005; Yeung et al., 2007; Choi et al., 2014). If these potassium channels are present on RGCs, application of Flu could lead to a lower resting membrane potential and a decrease in excitability, which would explain the observed effects. In line with this hypothesis, unpublished data from our lab shows that increasing RGC excitability by raising the extracellular potassium concentration leads to lower ChR2 stimulation thresholds. Why 18BGA should also increase stimulation thresholds is not clear, but as well as blocking gap junctions it affects a number of other ion channels (Rozental et al., 2001; Juszczak and Swiergiel, 2009) and possibly one of these effects is responsible for the increase in thresholds. 18BGA is also apparently cytotoxic at high concentrations (above 75 μM; Rozental et al., 2001), which might explain the sharp increase in threshold between 40 μM and 80 μM.

MFA has mixed effects on stimulation efficiency of ChR2-expressing RGCs. Like 18BGA and Flu, it increases thresholds in a dose-dependent manner, possibly also through the effect on the Kv7 channel that it shares with Flu (Yeung et al., 2007; Choi et al., 2014). Unlike the other two drugs, at 40 μM it increases the total number of cells with detectable responses. This increase may simply be due to unmasking of weak responding cells that could not be distinguished above the spontaneous activity. If, however, this increase in responders is a specific pharmacological effect of MFA, one possibility is that blocking gap junctions prevents ChR2 currents leaking into neighboring, non-ChR2-expressing RGCs, producing a stronger depolarization in the ChR2 RGC. In either case, the further increase in thresholds at 80 μM MFA seems to counteract the increase in responsiveness so that the number of responding cells returns to baseline. As such, moderate concentrations of MFA (similar to those used by Toychiev et al., 2013) seem to offer the best trade-off between improving SNR by decreasing spontaneous activity and not hindering ChR2 stimulation.

### 4.3. Spatiotemporally patterned stimulation of optogenetically sensitive retinas

The above analyses concern responses to wide-field, spatially homogenous flashes, which are not particularly perceptually interesting stimuli. Most visual tasks involve discerning information from a scene in which the pattern of light is varying in both space and time. Hence we also investigated, for the first time, the effects of decreasing spontaneous activity on spatiotemporally patterned optogenetic stimulation.

First, we mapped the receptive fields of ChR2-sensitive RGCs using spike-triggered averaging, or reverse correlation, of responses to small (2 × 2 or 4 × 4 pixels), brief flashes. Limitations of the stimulation device prevented the use of more typical reverse correlation stimuli, such as white noise (Chichilnisky, 2001), but nonetheless we were able to capture clear receptive fields in both control conditions and after blockade of spontaneous activity. The similarity of the recovered receptive fields in both conditions is more likely a testament to power of reverse correlation as a technique than evidence against the hypothesis that blocking spontaneous activity improves prosthetic responses. The recovered receptive fields were unipolar (as expected, since ChR2 is purely excitatory), slightly elliptical, and had an average diameter of 200–250 μm, which is on par with typical RGC dendritic arbor sizes (Sun et al., 2002). This is unsurprising: expression of ChR2 in the RGCs of this particular mouse line is throughout the cell membrane, including soma, dendrites, and axons (data not shown, see also Thyagarajan et al., 2010). One would thus expect the amount of depolarization caused by ChR2 stimulation to be proportional to the total cell surface area covered by the light stimulus, hence the response would be strongest when the light covers the soma and dendrites.

We also tested how well the population of optogenetically sensitive RGCs encodes spatiotemporal stimulus properties, namely motion direction of a moving bar. To measure this, we used Bayesian classification, which provides a lower bound on the information carried by a neural response about a stimulus (Quian Quiroga and Panzeri, 2009) and has been used previously to compare different retinal coding strategies to behavioral performance (Jacobs et al., 2009). The decoder was based on each cell's time to peak firing after stimulus onset, which in the absence of noise should correspond to when the bar crosses the cell's receptive field. It also took all cells as conditionally independent given the stimulus (the naive Bayesian assumption), which is plausible as the only stimulus-driven input each ChR2 RGC in a blind retina should receive is from the activation of ChR2 itself. Under these assumptions, the decoder performed much better when there was less spontaneous activity. This improvement was statistically significant for MFA, but not 18BGA or Flu. The lack of improvement for 18BGA and Flu might be because high concentrations of these drugs reduce the number of optogenetically responsive cells: many low SNR cells might encode motion direction just as well as a few high SNR cells. Flupirtine is effective at lower concentrations than 18BGA or MFA (Figure 6; see also Choi et al., 2014), so in one experiment we recorded moving bar responses at 0 and 20 μM Flu, but despite vastly reduced spontaneous activity, the decoder performance was virtually identical (data not shown). Thus, these data support that conclusion that blockade of spontaneous activity with MFA improves the encoding of spatiotemporal information available in optogenetically-evoked RGC responses. However, as we have only tested this using one set of stimuli, further investigation using different stimuli and encoding strategies will be needed to confirm this.

### 4.4. Implications for treatment of retinal degenerations

This study adds more evidence to the idea that reducing spontaneous hyperactivity in degenerate retinas could potentially improve the quality of vision returned by retinal prosthetics and that this is a worthwhile avenue to pursue in the search for improved treatments for retinal dystrophies such as RP. Further, by investigating a number of drugs we have provided information as to best choice of drug if a pharmacological strategy is chosen to reduce spontaneous activity in retinal prosthetic patients (but see below). Flu is an analgesic, anticonvulsant and muscle relaxant that is currently being investigated for possible neuroprotective effects (Friedel and Fitton, 1993; Klawe and Maschke, 2009; Szelenyi, 2013), MFA is a non-steroidal anti-inflammatory drug and analgesic (Juszczak and Swiergiel, 2009), and 18BGA is a flavoring agent derived from licorice (Asl and Hosseinzadeh, 2008). Thus, all three drugs are at least safe for human consumption and, in the case of Flu and MFA, already clinically approved drugs. However, given the apparent negative effects of 18BGA and Flu on ChR2 stimulation, MFA is probably the best candidate, at least where optogenetic retinal prostheses are concerned. There is some evidence that MFA is retinotoxic (Sun et al., 2013), but only at concentrations much higher than those used in this study. In particular, concentrations of around 40–50 μM seem to be effective at improving prosthetically-evoked responses without adverse effects either on optogenetic stimulation or the retina itself.

#### 4.4.1. How best to dampen spontaneous hyperactivity?

There may be problems with a pharmacological strategy to decrease spontaneous activity and improve prosthetic vision in a clinical setting. In particular, there is the challenge of delivering sustained, controlled, targeted dosages of the chosen drug to the retina. Systemic administration of both Flu and MFA can have a number of unpleasant side effects (Friedel and Fitton, 1993; Juszczak and Swiergiel, 2009; Klawe and Maschke, 2009; Szelenyi, 2013), as can excessive consumption of licorice, of which 18BGA is a metabolite (Asl and Hosseinzadeh, 2008; Juszczak and Swiergiel, 2009). These negative side-effects may not be a worthwhile trade-off, especially if the improvement in vision is modest. An alternative might be intravitreal injection, but if the chosen compound washes out in a matter of hours, as is the case for Flu and MFA *in-vitro*, then this would obviously be impractical. One solution might be to deliver the drug in the form of a slow-release compound that remains in the eye and releases the drug at a controlled rate, but this may be a considerable biomedical engineering challenge.

Fortunately, in this study we have shown that multiple drugs block spontaneous activity and improve SNR, independently of the mechanism of action. Extending this principle, it may be that non-pharmacological strategies to decrease spontaneous activity would also improve vision, while avoiding the challenges described above. For example, if increasing the potassium conductance of AII amacrine cells decreases spontaneous activity (Choi et al., 2014), then gene therapy to increase Kv7 potassium channel expression in AIIs or introduce a modified form of the channel with higher conductance might have a similar effect to Flu or MFA. Moreover, if the chosen promoter is selective for AIIs, then it would avoid the decrease in responsiveness observed with high concentrations of Flu. Genetic knock-out of gap junctions could also work (Ivanova et al., 2015), but it would be better to restrict the knock-out to AIIs rather than pan-retinally as gap-junctions play a number of important roles in vision (Bloomfield and Völgyi, 2009). Alternatively, in the case of an optogenetic retinal prosthesis, one could envisage expressing an inhibitory opsin with a distinct absorption spectrum, such as halorhodopsin, in the AIIs and using a steady background light to hyperpolarize them and dampen the oscillations. However, this would increase the power consumption of the prosthetic device.

#### 4.4.2. How much does the prosthetic strategy matter?

The results from this study, combined with the work of Toychiev et al. (2013) and Ivanova et al. (2015), suggest that blocking spontaneous activity to improve prosthetic vision works as a general strategy, somewhat independently of the means of vision restoration. It has now been shown to work for residual photoreceptor responses, electrical stimulation and optogenetic stimulation. Thus, this strategy should be effective for electrical prostheses, optogenetic prostheses and even non-prosthetic strategies such as strategies to halt photoreceptor death (e.g., Cuenca et al., 2014) or replace them with exogenously grown or endogenously regenerated photoreceptors (e.g., Jayakody et al., 2015). However, the method of vision restoration has some implications for the choice of strategy for block spontaneous activity. Blocking gap-junctions with MFA is fine for an epiretinal electrical prosthesis or RGC-targeting optogenetic process, whereby we assume the inner retina is incorrigibly degenerated and thus stimulate the RGCs directly, perhaps with the help of a retina-mimicking encoder (Nirenberg and Pandarinath, 2012). However, photoreceptor-preserving/replacing approaches, subretinal electrical prostheses and bipolar-cell targeting optogenetic prostheses all rely on an intact inner retina to encode the visual scene in a way the brain can understand. Setting aside the question of whether inner retinal function is preserved in retinal degeneration (Marc et al., 2003; Jones and Marc, 2005; Marc et al., 2007; but see also Busskamp et al., 2010), the AII amacrine cells (Farsaii and Connaughton, 2011) and gap junctions (Bloomfield and Völgyi, 2009) both have important roles in normal visual function and interfering with them to reduce spontaneous activity may corrupt the visual signal sufficiently to bring no net gain in quality of restored vision. Choi et al. (2014) suggest that restoration of photoreceptor light responses or light-sensitization of bipolar cells might bring the AIIs into a more depolarized, non-oscillating regime, but this depends on the amount of depolarization introduced by the light sensitization. Hence whether spontaneous activity reduction is beneficial in photoreceptor- or bipolar cell-targeting treatments remains an open question.

### 4.5. Light requirements

Retinal degenerate spontaneous hyperactivity has been largely overlooked in previous studies of optogenetic retinal prosthesis. One possible explanation for this is the light intensities used. Most reports are accompanied by dramatic rasters of light responses, where the peak in the PSTH towers above what appears to be an insignificant baseline firing rate (Bi et al., 2006; Lagali et al., 2008; Zhang et al., 2009; Busskamp et al., 2010; Doroudchi et al., 2011). The average irradiance at the level of the retina in this study was around 25 μW/mm^2^ or approximately 6.3 × 10^15^ photons/s·cm^2^ at 490 nm (the peak of the μLED array emission spectrum), which is at the lower end of light intensities typically used in studies of optogenetic retinal prostheses (but compare Lin et al., 2008; Cehajic-Kapetanovic et al., 2015; van Wyk et al., 2015; also, as noted in Section 2.4, the peak irradiance at the retinal level in this study will be higher, but not by more than half a log unit). The implications for this are two-fold. Firstly, being able to evoke visual responses with lower light intensities lowers the overall power requirements of the retinal prosthetic device. Moreover, if a prosthesis needs to drive the retina very strongly to produce a reliable percept, then this suggests that it will only be able to transfer information about high-contrast visual features, making them no better than currently available devices and necessitating image processing strategies to improve scene contrast (e.g., Al-Atabany et al., 2013). Decreasing the amount of spontaneous activity may allow lower-contrast visual features to be perceived, improving the dynamic range of retinal prosthetic devices. Ultimately psychophysical studies in prosthetic patients will be needed to determine whether this is indeed the case.

### 4.6. Conclusions

We have demonstrated that reducing spontaneous activity works as a strategy to improve the quality of optogenetically-evoked retinal responses, increasing the SNR of optogenetic responses and improving the ability to determine stimulus properties from RGC firing. Moreover, of the drugs tested so far, we have shown that MFA is the most promising in terms of decreasing spontaneous activity without hampering optogenetic stimulation. This provides important information and guidance for future research into improving the quality of vision returned by retinal prosthetics.

## Author contributions

JB designed the study, conducted all experiments, analyzed all data and wrote the paper. PD provided the microLED array and contributed to the writing of the paper. ES contributed to the design of the study and the writing of the paper.

## Funding

This work was supported by the Wellcome Trust [096975/Z/11/Z]. Additionally, development of microLED array was funded by the European Commission under the OptoNeuro project [FET-Open-249867].

### Conflict of interest statement

The authors declare that the research was conducted in the absence of any commercial or financial relationships that could be construed as a potential conflict of interest.
